# Formulation and Characterization of Mucoadhesive Buccal Films of Glipizide

**DOI:** 10.4103/0250-474X.40330

**Published:** 2008

**Authors:** Mona Semalty, A. Semalty, G. Kumar

**Affiliations:** Department of Pharmaceutical Sciences, H. N. B. Garhwal University, Srinagar (UA) - 246 174, India; 1 Department of Pharmacy, S. G. R. R. I. T. S. Patelnagar, Dehradun - 248 001, India

**Keywords:** Mucoadhesive, buccal film, glipizide, *in vitro* studies

## Abstract

Mucoadhesive buccal films of glipizide were prepared by solvent casting technique using hydroxypropylmethylcellulose, sodium carboxymethylcellulose, carbopol-934P and Eudragit RL-100. Prepared films were evaluated for weight, thickness, surface pH, swelling index, *in vitro* residence time, folding endurance, *in vitro* release, permeation studies and drug content uniformity. The films exhibited controlled release over more than 6 h. From the study it was concluded that the films containing 5 mg glipizide in 4.9% w/v hydroxypropylmethylcellulose and 1.5% w/v sodium carboxymethylcellulose exhibited satisfactory swelling, an optimum residence time and promising drug release. The formulation was found to be suitable candidate for the development of buccal films for therapeutic use.

Amongst the various routes of administration tried so far for novel drug delivery systems, localized delivery to tissues of the oral cavity has been investigated for a number of applications including the treatment of toothaches^1^, periodontal disease^2^,^3^, bacterial and fungal infections^4^, aphthous and dental stomatitis^5^ and in facilitating tooth movement with prostaglandins^6^. Over the last two decades mucoadhesion has become of interest for its potential to optimize localized drug delivery, by retaining a dosage form at the site of action (e.g. within gastrointestinal tract) or systemic delivery, by retaining a formulation in intimate contact with the absorption site (e.g. the buccal cavity). Mucoadhesion maybe defined as a state in which two materials, one of which is mucus or a mucous membrane, is held together for extended period of time^7^. Recently, Jasti *et al.* Salamat-Miller *et al.* and Semalty *et al.* has reviewed the use of mucoadhesive polymers in buccal drug delivery and highlighted the use of novel mucoadhesive polymers^8^–^10^. Various studies have been conducted on buccal delivery of drugs using mucoadhesive polymers. Attempts have been made to formulate various mucoadhesive devices including tablets^11^, films^12^, patches^13^,^14^, disks^15^,^16^, strips^17^, ointments^18^ and gels^19^. Buccal film may be preferred over adhesive tablet in terms of flexibility and comfort. In addition, they can circumvent the relatively short residence time of oral gels on the mucosa, which are easily washed away and removed by saliva. Moreover, the buccal films are able to protect the wound surface, thus reducing pain and treating oral diseases more effectively^20^.

Glipizide is a second generation sulfonylurea used as an antidiabetic agent. Glipizide is one of the most potent of the sulfonylurea antidiabetic agents. It is 100 times more potent than tolbutamide in evoking pancreatic secretion of insulin^21^. It differs from other oral hypoglycemic drugs in that tolerance to its action apparently does not occur. It also upregulates insulin receptors in the periphery, which seems to be the primary action. Its short biological half-life (3.4 ± 0.7 h) necessitates its administration in 2 or 3 doses of 2.5 to 10 mg per day. Moreover, about 90% of the drug is metabolized in the liver forming several inactive metabolites^21^. Thus an attempt has been made to develop a buccal mucoadhesive dosage form of glipizide for improving and enhancing bioavailability in a controlled release fashion. It may also be possible to circumvent the hepatic first pass effect by administering the drug through buccal mucosa.

The present work deals with the formulation and characterization of mucoadhesive buccal films of glipizide using mucoadhesive polymers like hydroxy propylmethylcellulose, Carbopol-934P, Eudragit RL-100 and sodium carboxymethylcellulose.

## MATERIALS AND METHODS

Glipizide was obtained as a gift sample from USV Ltd (Daman, India). Hydroxypropylmethylcellulose (HPMC-E15), Carbopol-934P (CP-934P), Eudragit RL-100 and sodium carboxymethylcellulose, 1500-400cps (SCMC) were procured from Central Drug House, Mumbai. Propylene glycol was procured from E. Merck (P) Ltd, Mumbai. All other reagents used were of analytical grade. The films were prepared by solvent casting method.

### Preparation of mucoadhesive buccal films:

Buccal films of glipizide were prepared by solvent casting technique employing aluminum foil cups (placed on glass surface) as substrate^22^. Composition of a single circular cast film of various formulations is mentioned in Table 1. Buccal films were prepared by using HPMC-E15 alone and in combination with CP-934P, Eudragit RL-100 and sodium CMC (high viscosity grade). Propylene glycol, a plasticizer is used in the concentration of 30% w/w. Ethanol was used as a solvent.

**TABLE 1:** COMPOSITION OF MUCOADHESIVE BUCCAL FILMS

Ingredients	Formulations			
	
	F1	F2	F3	F4
Glipizide(g)	0.30	0.30	0.30	0.30
HPMC-E15 (g)	1.30	1.00	1.00	1.00
Sodium CMC-H (g)	-	0.30	-	-
Eudragit RL100 (g)	-	-	0.30	-
Carbopol -934P (g)	-	-	-	0.30
Propylene Glycol (ml)	0.48	0.48	0.48	0.48
Ethanol (95%) ml	20	20	20	20

The calculated amounts of polymers were dispersed in ethanol. Three hundred milligrams of glipizide was incorporated in the polymeric solutions after levigation with 30% w/w propylene glycol which served the purpose of plasticizer as well as penetration enhancer. The medicated gels were left overnight at room temperature to obtain clear, bubble-free gels. To prevent the evaporation of alcohol, medicated gels were filled into the vials and closed tightly by the rubber closures. The gels were caste into aluminum foil cups (4.5 cm diameter), placed on a glass surface and allowed to dry overnight at room temperature (25°) to form a flexible film. The dried films were cut into size of 20 mm diameter, packed in aluminum foil and stored in a desiccator until further use.

### Film weight and thickness:

For evaluation of film weight three films of every formulation were taken and weighed individually on a digital balance (Fisher Brand PS-200). The average weights were calculated. Similarly, three films of each formulation were taken and the film thickness was measured using micrometer screw gauge (Mitutoyo MMO-25DS) at three different places and the mean value was calculated.

### Surface pH of films:

For determination of surface pH three films of each formulation were allowed to swell for 2 h on the surface of an agar plate. The surface pH was measured by using a pH paper placed on the surface of the swollen patch. A mean of three readings was recorded^20^.

### Percent swelling:

After determination of the original film weight and diameter, the samples were allowed to swell on the surface of agar plate kept in an incubator maintained at 37±0.2°. Increase in the weight of the films (*n* = 3) was determined at preset time intervals (1-5 h). The percent swelling, %S, was calculated using the following equation: Percent Swelling (%S) = (X_t_ – X_o_/X_o_) × 100, where X_t_ is the weight of the swollen film after time t, X_o_ is the initial film weight at zero time^23^.

### Folding endurance:

Three films of each formulation of size (2 × 2 cm) were cut by using sharp blade. Folding endurance was determined by repeatedly folding a small strip of film at the same place till it broke. The number of times, the film could be folded at the same place without breaking gave the value of folding endurance. The mean value of three readings and standard deviation were shown in Table 2.

**TABLE 2:** PHYSICAL EVALUATION OF MUCOADHESIVE BUCCAL FILMS OF GLIPIZIDE

Form. Code	Surface pH	Thickness (mm)	Swelling Index (2h) Without drug	Swelling Index (2h) With drug	*In vitro* Residence time (h)	Folding Endurance	Content Uniformity (mg/20 mm diameter of the film)
F1	6.59 ± 0.02	0.28 ± 0.03	40.0 ± 0.3	46.4 ± 0.33	2.25 ± 0.56	164.6 ± 4.53	4.64 ± 0.006
F2	6.50 ± 0.02	0.25 ± 0.03	41.4 ± 1.6	46.8 ± 1.25	3.00 ± 0.72	234.3 ± 12.66	4.66 ± 0.045
F3	6.42 ± 0.03	0.26 ± 0.01	15.9 ± 0.7	17.2 ± 1.16	1.75 ± 0.91	207.7 ± 11.37	4.82 ± 0.014
F4	6.15 ± 0.10	0.26 ± 0.02	29.3 ± 1.0	32.0 ± 0.26	4.00 ± 0.10	290.0 ± 4.0	4.66 ± 0.062

(*n* = 3) Abbreviations used: HPMC-hydroxypropylmethylcellulose; Sodium CMC-H-Sodium carboxymethylcellulose-high viscosity grade

### *In vitro* residence time:

The *in vitro* residence time was determined using IP disintegration apparatus. The disintegration medium was 800 ml of pH 6.6 phosphate buffer (PB) maintained at 37±2°. The segments of rat intestinal mucosa, each of 3 cm length, were glued to the surface of a glass slab, which was then vertically attached to the apparatus. Three mucoadhesive films of each formulation were hydrated on one surface using pH 6.6 PB and the hydrated surface was brought into contact with the mucosal membrane. The glass slab was vertically fixed to the apparatus and allowed to move up and down. The film was completely immersed in the buffer solution at the lowest point and was out at the highest point. The time required for complete erosion or detachment of the film from the mucosal surface was recorded (*n* = 3) as given in Table 2.

### Drug content uniformity:

Three film units (each of 20 mm diameter) of each formulation were taken in separate 100 ml volumetric flasks, 100 ml of pH 6.6 phosphate buffer was added and continuously stirred for 24 h. The solutions were filtered, diluted suitably and analyzed at 274 nm in a UV spectrophotometer (Thermospectronic UV-1). The average of drug contents of three films was taken as final reading.

### *In vitro* release study:

The USP XXIV six station dissolution apparatus type 1 (V Scientific Model No. DA-6DR) was used throughout the study. One film of each formulation was fixed to the central shaft at just above the basket, using a cyanoacrylate adhesive. The dissolution medium consisted of 900 ml pH 6.6 phosphate buffer (PB). The release study was performed at 37 ± 0.5° with a rotation speed of 50 rpm. The release study was carried out for 6 h. After every hour, 1 ml sample was withdrawn from each station and the same volume was replaced (with the dissolution medium) back to the stations. Each withdrawn sample was filtered, diluted suitably and then analyzed spectrophotometrically at 274 nm. The data presented were the mean of three determinations.

### *Ex vivo* permeation studies:

In this study, porcine buccal mucosa was used as a barrier membrane. The buccal pouch of freshly sacrificed animal was procured from local slaughter house. The buccal mucosa was excised and trimmed evenly from the sides. It was then washed in isotonic phosphate buffer (pH 6.6) and used immediately^24^.

The *ex vivo* permeation studies of mucoadhesive buccal films of glipizide through an excised layer of porcine buccal mucosa were carried out using the modified Franz diffusion cell^23^. A 2.0 cm diameter film of each formulation under study was placed in intimate contact with the excised porcine buccal mucosa and the topside was covered with aluminum foil as a backing membrane. Teflon bead was placed in the receptor compartment filled with 100 ml of pH 7.4 phosphate buffer. The cell contents were stirred with a magnetic stirrer and temperature of 37±1° was maintained throughout the experiment. The samples were withdrawn at every hour, filtered, diluted suitably and then analyzed using UV- spectrophotometer at 276 nm.

## RESULTS AND DISCUSSION

Mucoadhesive buccal films of glipizide were prepared using mucoadhesive polymers HPMC-E15, CP-934P, Eudragit RL-100 and sodium CMC. Propylene glycol was used as the plasticizer as well as penetration enhancer. The drug delivery system was formulated as a matrix. The films were characterized for their physical characteristics, bioadhesive performance, release characteristics, surface pH, thickness, folding endurance, drug content uniformity and percent swelling (Table 2). The film thicknesses were observed to be in the range of 0.245±0.028 mm to 0.282±0.032 mm and weight was found to be in the range of 56±1.86 mg to 84±0.74 mg.

Considering the fact that acidic or alkaline pH may cause irritation to the buccal mucosa and influence the degree of hydration of polymers, the surface pH of the buccal films was determined to optimize both drug permeation and mucoadhesion^25^,^26^. Attempts were made to keep the surface pH as close to buccal/salivary pH as possible, by the proper selection of the polymers for developing the buccal films. The surface pH of all the films was within the range of salivary pH. No significant difference was found in surface pH of different films.

Hydration is required for a mucoadhesive polymer to expand and create a proper macromolecular mesh of sufficient size, and also to induce mobility in the polymer chains in order to enhance the interpenetration process between polymer and mucin. Polymer swelling permits a mechanical entanglement by exposing the bioadhesive sites for hydrogen bonding and/or electrostatic interaction between the polymer and the mucous network^27^. However, a critical degree of hydration of the mucoadhesive polymer exists where optimum swelling and bioadhesion occurs^28^. The effect of glipizide on the swelling behaviour and the residence time of various mucoadhesive polymers, was also observed (Table 2). The medicated films showed high swelling index in comparison to plain films. The addition of the water-insoluble drug increased the water uptake of the film. This is possibly due to micronized drug particles which exist between the polymer chains allowing each chain to hydrate freely, resulting in weak hydrogen bonding areas around the glipizide molecules. These areas may increase the strength of the swollen layer followed by an obvious increase in the amount of penetrated water^29^. Indomethacin, a practically water-insoluble drug, was found to increase the swelling behaviour of HPMC matrices^29^, while lower swelling indices were observed when the same drug was added to Gantrez-169 compressed matrix^30^. The influence of drug on the swelling properties of polymer matrices is primarily dependent on the substituted groups of the polymer. The hydroxyl group in the molecules plays an important role in the matrix integrity of the swollen hydrophilic cellulose matrices. The amount and properties of the incorporated drug determine matrix integrity.

The comparative percentage swelling for various formulations was in order of F2 > F1 > F4 > F3. The percentage swelling of HPMC-E15 films was reduced by the addition of Carbopol 934P and Eudragit-RL100 and increased by the addition of SCMC. SCMC containing films showed higher percent swelling due to presence of more hydroxyl group in the SCMC molecules. The water-soluble hydrophilic additive dissolves rapidly resulting in high porosity. The void volume is thus expected to be occupied by the external solvent diffusing into the film and thereby accelerating the dissolution of the gel^31^.

The incorporation of the drug induced significant reduction of the residence time of various formulations. The enhanced erosion rate was observed with the non-ionic polymers (HPMC with Eudragit RL100). As the particle swells, the matrix experiences intra-matrix swelling force which promotes disintegration and leaching of the drug leaving behind a highly porous matrix. Water influx weakens the network integrity of the polymer, thus influencing structural resistance of the swollen matrices, which in turn results in pronounced erosion of the lose gel layer^30^. The water-soluble hydrophilic polymers like SCMC dissolve rapidly and introduce porosity. The void volume is thus expected to be occupied by the external solvent which diffuses into the film and thereby accelerate the dissolution of the gel^31^. *In vitro* residence time of the film was in the order of F4 > F2 > F1 > F3. The folding endurance was measured manually, by folding the film repeatedly at a point till they broke. The breaking time was considered as the end point. Folding endurance was found to be highest for F4 (290 ± 4.0) and lowest for F1 (164.55±4.527). It was found that folding endurance of HPMC films was increased by the addition of polymers in the order; Eudragit-RL100 SCMC > Carbopol 934P. The folding endurance values of the films were found to be optimum and therefore the films exhibited good physical and mechanical properties.

Drug content in formulations was uniform with a range of 4.64±0.006 mg/20 mm diameter of the film (F1) to 4.82±0.016 mg/20 mm diameter of the film (F3). On this basis, it was found that the drug was dispersed uniformly throughout the film.

**Fig. 1:**
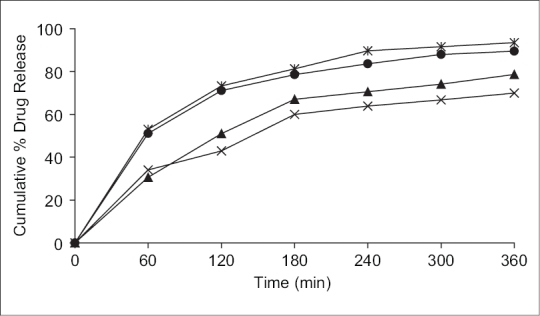
Cumulative percent drug release in pH 6.6 phosphate buffer. Formulation F1 (–●–), F2 (–✳–), F3 (–×–), F4 (–▲–).

*In vitro* release studies of various formulations were performed using pH 6.6 phosphate buffer as dissolution medium and measuring drug concentration spectrophotometrically at 274 nm. Distinguishable difference was observed in the release of glipizide films containing eudragit, carbopol and SCMC in the graph plotted between the cumulative per cent drug released from the formulations and the time (fig. 1). During dissolution, SCMC containing films swelled forming a gel layer on the exposed film surfaces. The loosely bound polymer molecules in these films were readily eroded, allowing the easy release of glipizide as compared to Eudragit RL-100^32^. After 6 h the release was found to be 89.50, 93.45, 69.89 and 78.65% in formulation F1, F2, F3 and F4, respectively (fig. 1).

SCMC and carbopol polymers exhibited high swelling, the film weight of these polymers was noted to be increased to the extent of 25 to 60% from the initial weight within 2 h (Table 2). Although the marked increase in surface area during swelling can promote drug release but the increase in diffusion path length of the drug may paradoxically delay the release. In addition, the thick gel layer formed on the swollen film surface is capable of preventing matrix disintegration and controlling additional water penetration^33^. SCMC films showed high dissolution rate as compared to Eudragit RL100 films. It was found that the drug release from the films varied with respect to the proportion of polymers. Preliminary studies done with the groups of formulations, from which these four formulations were selected, showed that increase in the polymer concentration reduced the diffusion of drug from the matrix. Amongst all formulations, formulation F2 showed the good release pattern as compared to others.

**Fig. 2:**
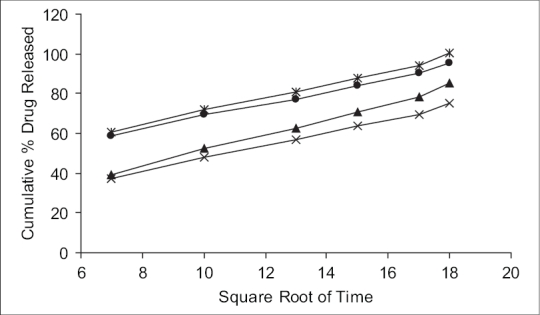
Higuchi's diffusion plot for different formulations. Formulation F1 (–●–), F2 (–✳–), F3 (–×–), F4 (–▲–).

Mechanism of drug release whether diffusion, swelling or erosion was confirmed by Higuchi's plots. fig. 2 shows the graphical representation of cumulative percentage drug release versus square root of time. The Higuchi's Plots were found to be linear with correlation coefficient values of 0.995, 1.037, 0.840 and 0.922 for F1, F2, F3 and F4, respectively. It was concluded that the release of drug from the films followed the diffusion controlled mechanism in all the formulations.

**Fig. 3:**
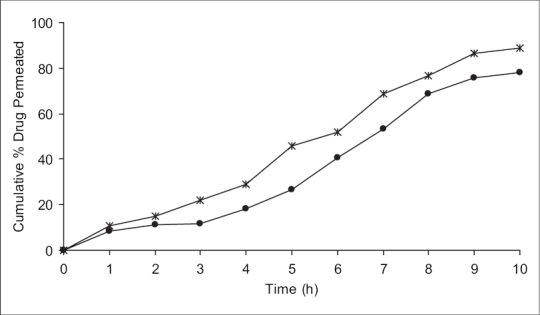
*Ex vivo* permeation studies of selected mucoadhesive buccal films of glipizide. Permeation studies in pH 7.4 phosphate buffer of formulations F1 (–●–) containing HPMC 6.3% w/v and F2 (–✳–) containing 4.9% w/v HPMC and 1.5% w/v SCMC with 5 mg glipizide in each film.

It was also concluded that formulation F1 (containing HPMC alone) and F2 (containing HPMC with SCMC) showed good swelling, a convenient residence time as well as promising drug release pattern. On the basis of release pattern, swelling and residence time, F1 and F2 formulations were selected for *ex vivo* study. In *ex vivo* study, drug permeation through the porcine buccal mucosa was determined for formulation F1 and F2 (fig. 3). The drug permeation was found to be 78.25% and 89.01% in F1 and F2 after 10 h.

The present study indicates a good potential of erodible mucoadhesive buccal films containing glipizide for systemic delivery with an added advantage of circumventing the hepatic first pass metabolism. The results of the study show that therapeutic levels of glipizide can be delivered buccally. It may be concluded that the films containing 5 mg glipizide in 4.9% w/v HPMC with 1.5% w/v SCMC (F2), show good swelling, a convenient residence time and promising controlled drug release, thus seems to be a potential candidate for the development of buccal film for effective therapeutic use. *In vivo* studies need to be designed and executed to substantiate further *in vitro - in vivo* correlation.
